# Business model innovation at the bottom of the pyramid – A case of mobile money agents

**DOI:** 10.1016/j.jbusres.2021.01.029

**Published:** 2021-04

**Authors:** Nkemdilim Iheanachor, Yinka David-West, Immanuel Ovemeso Umukoro

**Affiliations:** Lagos Business School, Pan-Atlantic University, Ajah, Lagos, Nigeria

**Keywords:** Business model, Business model innovation, Digital financial services, Financial services agents, Financial inclusion, Nigeria

## Abstract

Business models have historically facilitated the ability of firms to create and capture value. Focusing on financial service agents (FSAs) as actors in the Nigerian financial services industry, this study helps to elucidate how value creation and distribution can facilitate business model innovation (BMI) in an emerging market. We deployed Osterwalder and Pigneur’s business model canvas alongside Amit and Zott’s Sources of Value in e-Business (SVCeB) model in mapping FSA business models and value creation sources. We find that the constant need to align the resources of a firm with the demand conditions at the customer end triggers the need for BMI by FSAs. The findings also demonstrate that FSAs have weak business models that inhibit their sustainability and ultimately impede their ability to play their role in closing the country’s financial exclusion gap. We suggest the need for business model innovation by FSAs as a pathway to viability, profitability and sustainability.

## 1 Introduction

Many organizations in emerging markets are increasingly experiencing difficulties in conducting their businesses traditionally. These organizations are further challenged by the aim to grow and the difficulty in extending their businesses to new locations and jurisdictions that do not have viable and economically empowered consumers upon which to build traditional models. Sustaining competitive advantage has become increasingly difficult for emerging market firms that seek to extend their services to new locations.

This issue has made the phenomenon of business model innovation (BMI) an important topic of discourse for academics and practitioners alike in the search to extend such services to new locations and to identify new paths for sustainability and competitive advantage. Despite efforts to define BMI, our collective understanding of the phenomenon remains vague. In addition to lacking a defined process for innovating a business model, its application by researchers has been more widespread in examining approaches deployed by organizations in generating new demand for existing products or delivering additional benefits to existing customers (McGrath, 2010, Smith et al., 2010). This study aims to contribute to the body of knowledge on BMI by examining the phenomenon in the context of the creation and delivery of financial services in an emerging market characterized by poor consumers and severe resource constraints.

In Nigeria, as of 2018, approximately 36.8% of the adult population, representing 36.6 million individuals, was excluded from formal financial services (EFInA, 2018). The business model of serving the excluded population through brick-and-mortar commercial bank branches is prohibitive due to the associated high costs. The high associated cost of serving the poor has resulted in the creation of new business models that create and deliver new financial service value propositions and avoid the high associated costs of deploying the infrastructure and resources of brick-and-mortar commercial bank branches. The most commonly adopted medium for the delivery of financial services to the poor is that of digital financial services (DFS).

DFS have proven to be the most critical enabler of access to formal financial services, with agent banking as the closest route to closing the financial exclusion gap, especially at the bottom of the pyramid. Notwithstanding this development, the financial services agents (FSAs) that are the drivers of agent banking are largely unprofitable despite the huge market that depends on their services. This is because these agents currently deploy business models that are not sustainable, resulting in high failure and churn rates. The need for financial services agents to innovate their business models to remain profitable and sustainable has thus become critical. This study adopts a case study approach to understand FSA business models and to make recommendations on business model innovation practices that will result in the profitability and viability of FSAs.

Despite the numerous benefits of financial inclusion, the Demirguc-Kunt, Klapper, Singer, Ansar, and Hess (2018) reports that, globally, 31% of the world’s population, representing approximately 1.7 billion adults are financially excluded (without access to any form of account). The implication is that these financially excluded individuals are unable to contribute to the formal economy in dealing with financial shocks as they do not have the leverage to fall back to when they are hit. The important issue of financial inclusion includes the ability to lift individuals out of poverty by enabling them to save, build an economic history that is useful for accessing credit, fund their children’s education, and provide access to quality healthcare. Although financial inclusion plays a critical role in enhancing sustainable development, poverty reduction, and promoting shared prosperity, poor, young, and small firms face the most significant barriers in terms of access to finance (Aguera, 2015). Consequently, the Universal Financial Access (UFA) initiative sought to reduce the number of unbanked and underbanked adults in the financial system to 1 billion by 2020.

While the deployment of alternative channels such as online banking, mobile banking, payment cards, ATMs, and POS has given rise to a digital financial service (DFS) ecosystem (David-West et al., 2019a, David-West et al., 2019b), serving the low-income segment at the bottom of the pyramid (BoP) would require alternative cost-effective delivery channels that are different from the traditional approach. This is because the cost of establishing physical bank branches in less economically viable locations deters banks from expanding to those areas. Other causes that characterize the bottom of the pyramid and the informal sector that might impede the expansion of financial access to the unbanked and underbanked include poor road networks, insecurity, the cost of mobilizing technological infrastructure, low literacy and low levels of formal education.

While various developments in the financial services industry have previously been studied, business models and their innovation – in particular among financial service agents in emerging market contexts – have received very little attention in the literature. Although the use of agents for delivering financial services to low-income consumers has proven to be a viable service delivery alternative, there are challenges. First, the critical mass of bankable consumers required for agents to be profitable is often not attained due to widespread poverty and the low income of target customers. These financial service consumers are also very price sensitive and may not be willing to pay the service fees required for such financial transactions. Based on an analysis of thirty cases drawn from the Nigerian financial services industry, this paper enhances our understanding of business model innovation by FSAs. Specifically, the study attempts to (a) identify the current business models of financial services agents and their sources of value creation, (b) investigate the extent to which financial services agents (FSAs) innovate their business models towards business sustainability, and (c) identify the current weaknesses and innovation opportunities in the business models of financial services agents.

This study was guided by three research questions: (1) What are the current business models deployed by financial services agents in Nigeria? (2) What are the sources of value creation of financial service agents? and (3) To what extent do financial services agents innovate their business models to drive scale and profitability?

This study enhances the understanding of BMI in financial services industries in emerging markets characterized by severe resource constraints. We applied a qualitative research design to investigate the impact of the business models of FSAs as well as their sustainability and ability to create and deliver value. The enhanced understanding of BMI in the context of FSAs in an emerging market can also help to explain BMI in other contexts.

## 2 Literature review

### 2.1 Business models and business model innovation

The term business model has been defined by different scholars to represent different perspectives depending on a firm’s objectives. Di Tullio, Valentinetti, and Rea (2018) report that one of the aspects of the business model concept that has received much debate is its definition. Given the role of the business model, several scholars have defined and classified it in many ways. Table 1 presents the broad nature of business model conceptualizations.

**Table 1:** Business model conceptualization.

Business Model Conceptualization	Definition	Literature source
Description	A *set* of choices, decisions, resources, and actions that are performed to create business value	Afuah, 2004, Aversa et al., 2015, Baden-Fuller and Morgan, 2010, Casadesus-Masanell and Heilbron, 2015, Girotra and Netessine, 2014
	A *description* of a firm’s roles, responsibilities, resources, and relationships	Cosenz, 2017, Casprini et al., 2014, Fielt, 2014, Markides, 2015, Osterwalder and Pigneur, 2010, Morris et al., 2006

Structure	*An architecture* or *system* that aggregates resources for business success	Al-Aali and Teece, 2013, Amit and Zott, 2012, Baden-Fuller and Haefliger, 2013, Chatterjee, 2013, Kavadias et al., 2016, Kohler, 2015, Page and Spira, 2016, Teece, 2010, Timmers, 1998, Zott and Amit, 2010.
	An *architecture* of the product, service, and information flows including a description of the various business actors and their roles and a description of the potential benefits for the various business actors as well as a description of the sources of revenue	Mazzarol et al., 2018, Timmers, 1998

Unit of Analysis	A *method* of harnessing a firm’s resources to better serve customers and remain competitive and sustainable	Afua and Tucci (2003)
	A *system* of independent activities that transcend the focal firm and spans its boundaries	Amit and Zott, 2012, Zott and Amit, 2010
	A *business articulation* that provides an understanding of a firm’s activity portfolio	Demil and Lecocq (2010)

Design	A *design* that enables a firm converts a given set of strategic choices relating to markets, customers, and value propositions into value for commercial purposes	Arend, 2013, Shafer et al., 2005, George and Bock, 2011, Smith et al., 2010

Resource	A *set* of capabilities that is configured to enable value creation consistent with either economic or social strategic objectives.	Seelos and Mair (2007)

The different business model conceptualizations point to the roles and functions of creating commercial value for both customers and stakeholders. A business model can therefore be understood as a carefully conceived methodology or framework involving the why, how, what, and when of a firm’s business undertakings. It helps firms identify approaches to product or service development, deployment, and pricing aimed at meeting firm value propositions (Osterwalder & Pigneur, 2010). The business model must answer Peter Drucker’s age-old questions: *Who is the customer? What does the customer value? How does a firm earn a profit? What is the underlying economic logic that explains how a firm can deliver value to customers at an appropriate cost* (Blahová, 2017)?

Zott, Amit, and Massa (2011) note that the significance of business models to businesses can be seen in the central role they play in explaining firm performance. Business models help explain firm competitiveness by highlighting and defining the approaches and methods by which a firm builds and uses its resources to offer its customer better value and to make money in doing so (Di Tullio et al., 2018, Afua and Tucci, 2003, Afuah and Tucci, 2001). A business model can foster the integration of value capture and value creation within a firm if it is used as a unit of analysis in strategic management research with its traditional emphasis on value capture (Demil et al., 2015, Amit and Zott, 2012, Zott and Amit, 2007, Chesbrough and Rosenbloom, 2002). Demil et al. (2015) further posit that business models favour a holistic understanding of differential firm performance (strategy field) and opportunity development (entrepreneurship field) by focusing on the overall pattern of activities that explain *how* firms conduct business. The preceding business model definitions highlight the fact that such a model consists of several components across different areas of practice and organizational strategic intent.

A critical analysis of business model definitions and components shows that there is no universal standpoint across the business model literature regarding the elements that constitute a business model. While different elements may make up a business model, there are variations across different models depending on the objective and nature of the business. Some of these components are presented in Table 2.

**Table 2:** Components of a business model.

Business Models Components	Literature
Customer segments, value proposition, customer relationships, channels, key resources, key activities, key partners, cost structure, and revenue streams	Osterwalder and Pigneur (2010)
Customer benefits, configuration, and company frontiers	Hamel (2000)
Market opportunity, capabilities, and value	Applegate (2001)
Mission, structure, processes, revenues, legal issues, and technology	Alt and Zimmerman (2001)
Strategic choices, value creation, value capture, and the value network	Shafer et al. (2005)
Value proposition, the value creation and delivery system, and the value capture system	Richardson (2008)
Value proposition, value constellation, and profit maximization	Yunus et al. (2010)
Complementarity, efficiency, novelty, and lock-in	Amit and Zott (2001)
Value proposition, value creation and delivery, and value capture	Bocken et al. (2014)

A critical evaluation of the business model literature suggests that some key elements are present across a range of business models. These include value proposition, activities, customer segment or focus, resources and capabilities, partners/stakeholders, and revenue. This further confirms McNally (2013) definition of a business model as encompassing both internal (infrastructure and processes of creating efficiencies) and external (customers and processes of value creation) factors that are required to meet a firm’s objective.

### 2.2 Business model innovation

Demil and Lecocq (2010) describe BMI as a vehicle for corporate transformation and renewal. Zott and Amit (2010) note that there is an increasing consensus that BMI is central to a firm’s performance. Business model innovation refers to the discovery of a primarily different business model in an existing business (Markides, 2006). This definition highlights the fact that a firm’s current business model may no longer be appropriate for sustaining the existing business or robust for the firm’s new strategic imperatives. Similarly, Casadesus-Masanell and Zhu (2013) define BMI as the search for a firm’s new business logic and new ways to create and capture value for its stakeholders. Both definitions underscore the alignment of a firm’s business model to fit new market realities, either from a demand- or supply-side perspective. Similarly, Johnson, Christensen, and Kagermann (2008) posit that BMI is critical when there are significant market changes in key areas of a firm’s business model. Such changes may necessitate the need to respond to a shifting basis of competition and to build barriers against low-end disrupters and present opportunities to leverage new technologies, to adopt a job-to-be done focus where none previously existed, and to capture new segments using disruptive innovations (Johnson et al., 2008).

Overall, a firm’s existing business model would need to be altered or even abandoned to evolve a new model that can help deliver a firm’s strategy. However, Chesbrough, 2010, Chesbrough, 2007 warns that to innovate a firm’s business model, executives must first understand the existing business model and examine what paths exist for them to improve on. Giesen, Berman, Bell, and Blitz (2007) identified three types of BMI: industry models (innovations in the industry supply chain), revenue models (innovations in how companies generate value), and enterprise models (innovations in the role played by the structure of an enterprise in new or existing value chains). While business models are traditionally concerned with firm-level value creation and capture, business model innovation also raises questions about novelty in customer value propositions and the logical reframing and structural reconfiguration of firms (Spieth, Schneckenberg, & Ricart, 2014).

An understanding of the role-play definition and the various conceptualizations of the business model can facilitate business model innovation in financial services. Consequently, the result of BMI (and renovation as the case may be) must provide a better explanation to financial service providers on how a business should respond to the identified needs of target customer segments. It also should provide an approach to generating revenue at a reasonable cost while incorporating assumptions about how the business will both create and capture value (Gambardella & McGahan, 2010). Additionally, BMI should enact a commercial opportunity (George & Bock, 2011) and provide logic for creating value (Amit and Zott, 2015, Linder and Cantrell, 2000). Furthermore, it should contribute to the firm’s business sustainability. Zott et al. (2011) note that the survival of firms also depends on their ability to innovate new business models that are capable of both value creation and value capture within a value network of suppliers, partners, distribution channels and coalitions that span beyond a firm’s resources.

### 2.3 Business model innovation in financial services

Financial service providers (FSPs) that rely on conventional brick-and-mortar branches face stiff competition, especially with the emergence of new providers that rely on lower-cost electronic channels deployed in the delivery of financial services. There is also an influx of nonbank financial service providers in the form of financial technology firms (FinTechs) that are constantly disrupting the norm in terms of how financial services are provided to consumers. Given this condition, the need to reconfigure existing business models has received growing attention. First, business models help FSPs identify approaches to product or service development, deployment, and pricing aimed at creating and delivering a firm’s value proposition (David-West, Iheanachor, et al., 2019) in a frugal manner (David-West, Iheanachor, & Umukoro, 2019). However, unlike other sectors, the literature remains sparse on BMI in the financial services ecosystem. In advancing the concept of BMI in financial services, David-West, Iheanachor, et al. (2019 & 2016) argue that the concept has achieved prominence based on the fact that existing business models might not have yielded the desired organizational impact in the short-, mid- or long term, thus making such businesses unsustainable in the long term. To address this challenge, the need for BMI becomes a necessity.

For financial service agents, the need to undertake business model innovation is even more critical given the characteristics of the bottom-of-the-pyramid customers served by FSAs. Bottom-of-the-pyramid customers typically have lower disposable income than more affluent customers and must be served by models that are lower in cost. While there is little evidence that documents BMI at this level, David-West et al. (2016) posit that to be sustainable, FSPs must exploit institutional resources, assets, and capabilities to build viable business models and businesses. This requires a reconfiguration of existing business models from generic to specialist (focused) or a combination of both – depending on the peculiarities of the target market segment and available resources. Business model innovation thus enables FSPs to meet new market demands and realities by re-evaluating and reconceptualizing their business objectives as well as their value creation logic and thereafter re-imagine customers’ perceptions of the firm’s value proposition (Bocken, Short, Rana, & Evans, 2014).

Although undocumented, anecdotal evidence suggests that FSAs can be innovative in how they conduct their businesses. For instance, many FSAs manage their liquidity by circulating cash from other businesses they operate just to meet customers’ cash demands. In other instances, FSAs may rely on traditional bank accounts when they run out of floats to retain customers and stay profitable. While they may not be able to use advanced and sophisticated marketing channels, they largely rely on word-of-mouth as well as leverage community networks for marketing and customer acquisition. There is also the incorporation of other business activities into existing businesses to remain profitable and sustainable. These practices can be related to the business model typology suggested by Cavalcante, Kesting, and Ulhøi (2011). It includes new venture creation, activities extension, and business modification through the replacement of previous activities with new ones and business termination, which could lead to the establishment of an entirely new business.

### 2.4 Agent banking and financial service agents

**Fig. 1:**
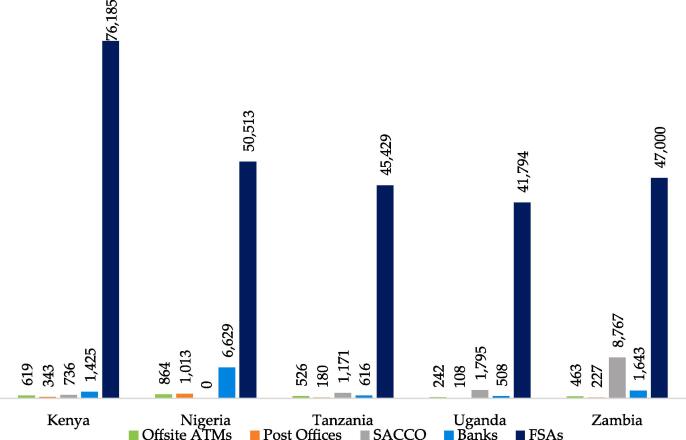
Financial services distribution channels across select African countries.

Financial service providers (FSPs) have begun reimagining their business models, especially in the area of distribution channels. In the past, traditional bank branches were the major means of providing financial services. However, the growing adoption of ubiquitous mobile technology has shifted emphasis from the establishment of brick-and-mortar branches to the launch of other low-cost channels such as financial services agents (FSAs) and self-service digital channels. A comparison of FSAs and other channels of distribution across select African countries (see Fig. 1) shows how significant FSAs are in FSP business models. The use of FSAs is on the rise as there are now over 3.7 million agents globally deployed by FSPs to deliver financial services to their customers.

The deployment of FSAs has been made possible through agent banking. Agent banking is a branchless banking arrangement that involves the use of agents and technology to transmit the details of financial transactions (Lauer, Dias, & Tarazi, 2011). David-West, Umukoro, et al. (2019) emphasized that this type of arrangement typically involves the use of mobile technologies to provide financial services to customers through agents instead of physical bank branches. This low-cost channel of financial service delivery is used all over the world, especially in developing countries where the cost of delivering financial services through physical bank branches is high. The Central Bank of Nigeria (2013) defines an agent as an entity engaged by a financial institution or nonbank financial institution to provide specific financial services on its behalf at the agents’ location. The agent is a third party who is authorized by the service provider or principal to act on its behalf and for whom the principal is liable concerning activities that are undertaken by the agent within the scope of its agent relationship or contract (Lauer et al., 2011, Tarazi and Breloff, 2011).

Although agent banking is seen as a cost-effective means of financial service delivery, its deployment does not automatically drive the adoption of financial services. Consequently, Demirguc-Kunt et al. (2018) notes that to encourage the adoption of digital financial services, service providers must tailor these services to the needs of disadvantaged groups mainly made up of women, poor people, and first-time users, who may have low literacy and numeracy skills, regardless of the technology driving these services. According to David-West et al., 2019b, Njunji, 2013, the costs associated with route-to-market and time-to-market pose challenges for low-income earners who will have to bear the cost-to-serve transferred to them as cost-to-use. As a result, the adoption and use of financial services delivered via branch locations have remained low among low-income earners. The agent banking arrangement is thus meant to eliminate or reduce such costs to enable low-income or geographically disconnected individuals to conveniently and affordably obtain access to financial services.

The deployment of FSAs requires FSPs to develop and manage their agent network or employ the services of independent superagents or agent aggregators based on a contractual arrangement. According to David-West, Umukoro, et al. (2019), agents exist at three levels: superagents, sole agents, and subagents. They note that while the superagent develops and manages a network of agents and sits at the apex of the agent pyramid, the sole agents are those who own and operate small franchises of agent outlets at strategic business locations akin to a small-scale superagent (SMSA) without requiring a superagent. However, the subagent is an individual who assumes agency on behalf of a financial service provider (FSP) by mediating between the FSP and the customer for commercial purposes.

While the superagent requires an operational licence from the apex bank, mainly the central bank, the sole agent and subagent are recruited by the superagent and must fulfil the requirements stipulated by the apex bank regulating the financial services offered at agent locations. Qualifications, conditions, and roles at all levels of agency vary from country to country (David-West et al., 2019b, Tarazi and Breloff, 2011). Consequently, these subagents (or agents) must undergo agent due diligence before they can provide financial services on behalf of their principals. Agent due diligence (ADD) helps reduce agent-related fraud activities and builds trust in a provider's brand (Davidson & McCarty, 2015; David-West, Umukoro, & Muritala, 2017; and Shi, 2011). Key activities carried out by sole agents and subagents (hereafter referred to as agents) include account opening, cash-in transactions, cash-out transactions, utility payments, remittances, loan disbursements, and others that may be permitted within agent banking guidelines (BMGF, 2013, David-West et al., 2019b, CGAP, 2009, Tarazi and Breloff, 2011, CGAP, 2012).

### 2.5 Agent banking models

Across several emerging markets, such as India, Nigeria, Paraguay, and Kenya, one of the qualifications for being considered to be an agent is ownership of an existing business such as a business centre, mom-and-pop shop, or provision store. Such a requirement is meant to ensure that the agent has a base on which to build the agency business and provide some form of leverage of business experience. This is further meant to help offset some of the fixed costs (rent, power etc.) that the agent will incur and might impact its profitability given that the needed scale might not be achieved at the onset. Agents of this nature are referred to as nondedicated agents. One of the advantages of this model is that it allows agents to cross-sell different products to existing customers in a complementary and efficient manner. According to Zott et al. (2011), this helps to create value for business as multiple services or products are bundled and provided together rather than delivering them separately. This helps enhance the utility customers derive from value propositions delivered by the agents. Opening an account at an agent location can also enable customers to pay utility bills. What is more, an agent would have successfully sold the utility payment service to a customer in place of an alternative channel.

In contrast, dedicated agents are those whose business scope is restricted to the provision of financial services. To be profitable with this model, agents must be located at an economically active location where banks are not present but are characterized by points of interest (POIs), such as schools, markets, hospitals, and motor parks, which are capable of generating economic activities. Businesses in these locations chiefly rely on agents to facilitate transactions. Another way to consider the agent banking business is to ascertain whether they operate based on an exclusive or nonexclusive model. An exclusive model confines an agent to serve only one financial service provider – usually, the FSP that signed and has invested in the development of the agent. A feature of this model is that the agent is not allowed to operate the terminals or devices of other FSPs. FSPs that run this agent banking model mainly do so to take control of the market share where their presence is most dominant. Conversely, the nonexclusive model allows agents to serve multiple FSPs, and as such, an agent may be signed by more than one FSP with interoperable platforms. By doing so, the agent can serve customers of other FSPs such as banks that may not have branches in those locations.

## 3 Theoretical models

Two theoretical models guided this study – Amit and Zott (2001) sources of value creation in e-business and Osterwalder’s business model canvas. These two models highlight the fundamental consideration for designing a business model that helps a firm realize its objectives.

### 3.1 Amit and Zott’s sources of value in e-Business (SVCeB)

This model focuses on how value is identified and where value can be created for both the firm and its customers. The model draws on different theoretical views including Schumpeter’s theory of creative destruction, strategic network theory and transaction cost economics, Porter’s value chain framework, and Barney’s resource-based view of the firm. Amit and Zott (2010) posit that new value can be created in e-business through the ways in which transactions are enabled. They postulate that the creation of value in an e-business lies in four interdependent dimensions: efficiency, complementarities, lock-in, and novelty. Given these interdependencies, they argue that no single entrepreneurship or strategic management theory can fully explain the value creation potential of e-business and further advocate the integration of the various theoretical perspectives on value creation. The elements of this model are as follows:

*Efficiency*: This source or value driver increases when the cost per transaction decreases. This implies that greater efficiency gains are enabled and result in more value as the cost is lowered. Amit and Zott (2001) reiterate that efficiency can be enhanced by reducing information asymmetry between consumers and suppliers through the use of low-cost e-channels for disseminating information as well as the delivery of services across virtual markets. The use of such low-cost channels in the financial service industry has become more evident among financial services providers. Channels such as POS, mobile banking, mobile money, and Internet banking have the potential to lower costs for providers while making access to financial services faster and easier. While the transaction process is substantially simplified and accelerated and costs are reduced, providers can benefit from mass adoption and high transaction volumes.

*Complementarities*: According to Amit and Zott (2001), complementarities create value for business when multiple services or products are bundled and provided together than when delivered separately. Customers derive more value from the utility of a product/service when purchased or used alongside another, which may be provisioned by another industry partner. Complementarities can be vertical (after sales support services) or horizontal (one stops purchasing). Amit and Zott (2001) emphasize that when customers access products/services that are complementary to the primary product of interest, efficiency may be enhanced as accessibility costs are reduced. For FSAs, the services of two or more FSPs may be delivered together on/through the same technological devices without necessarily acquiring different devices to serve the different providers. This can significantly reduce their CAPEX and OPEX.

*Lock-In*: This aspect prevents the migration of customers and strategic partners to competitors, thus creating value through repeat transactions, and it increases investment when partners have the incentive to maintain and improve their partnership with the firm (Amit & Zott, 2001). However, the firm’s value proposition must be compelling enough to retain both customers and partners. Lock-in may also be achieved by rewarding customer loyalty, service reliability, customer centricity, and brand improvement.

*Novelty*: A firm can create value through novel innovations. Novelty helps a firm build its brand and provides customers with a variety of products and services that competition may not. However, novelty requires a firm to have a strong pool of assets, resources, and capabilities. Amit and Zott (2001) posit that novelty can also help a firm bundle some products and services together, thereby providing customers with complementarities. Similarly, novelty can help a firm attract and retain customers, especially when such novelty has helped in building a strong brand. In essence, novelty has a strong relationship with lock-in. A firm’s business model must thus define the mechanisms through which novelty can be achieved to deliver on the value proposition for customers and partners while remaining profitable.

### 3.2 Osterwalder’s business model canvas (BMC)

The BMC identifies nine elements, which are as follows (see Table 3):

**Table 3:** Business model canvas elements.

S/No	Business Model Element	Description
1	Customer Segment	This is the building block that defines the groups of people or organizations an enterprise aims to serve. Such customers must form the heart of the business model. This term refers to the target market segment for which the business value is created (David-West, Iheanachor, et al., 2019).
2	Value Proposition	This is the economic and social value that solves a customer problem or satisfies a customer need (Chen et al., 2013, Osterwalder and Pigneur, 2011). Every value proposition comprises a selected bundle of products or services that caters to the requirements of a specific customer segment to attract and retain those customers. Good business value propositions must contain elements of value-innovation, which makes them distinct from those offered by competitors.
3	Channels	These refer to the mechanisms or modes of delivery through which customers are served. Channels serve as the conduit through which a firm relates to customers (service-oriented) or deliver goods to customers (product-oriented). Understanding market segments helps in the identification of the channels that are most appropriate in serving the customers (David-West, Iheanachor, et al., 2019).
4	Cost Structure	This element helps a business answer questions such as *what are the most significant cost inherent in the business* and *what avenues will be offset by those costs?* The operational efficiency of any business largely depends on how operating cost is structured.
5	Revenue streams	McNally (2013) identified a key question that every business must ask: *what are customers willing to pay for?* Once the appropriate customer needs have been identified, the revenue sources must then be factored into the business model.
6	Customer relationships	These are the types of relationships that are directly or indirectly maintained with customers (Chen et al., 2013). The nature of the relationship between firms and customers is crucial as it impacts customer acquisition and retention.
7	Key activities	These include the activities undertaken by a firm to deliver customer value proposition (Chen et al., 2013). Zeleti, Ojo, and Curry (2016) add that such activities describe the most important actions that a firm must take to make its business model work.
8	Key resources	Key resources include the technology, infrastructure, human resources, and other financials or non-financials that are vital in executing key activities (McNally, 2013, Chen et al., 2013) towards delivering the customer value proposition.
9	Key partners	These strategic networks are stable interorganizational ties that are strategically important to participating firms (Osterwalder & Pigneur, 2002). They extend beyond the firm’s entity, its customers and shareholders and include the value captured for key stakeholders, which requires a broader value-network perspective for innovating and transforming the business model (Beattie & Smith, 2013; Sommer, 2012; and Zott et al., 2011).

## 4 Methodology

**Fig. 2:**
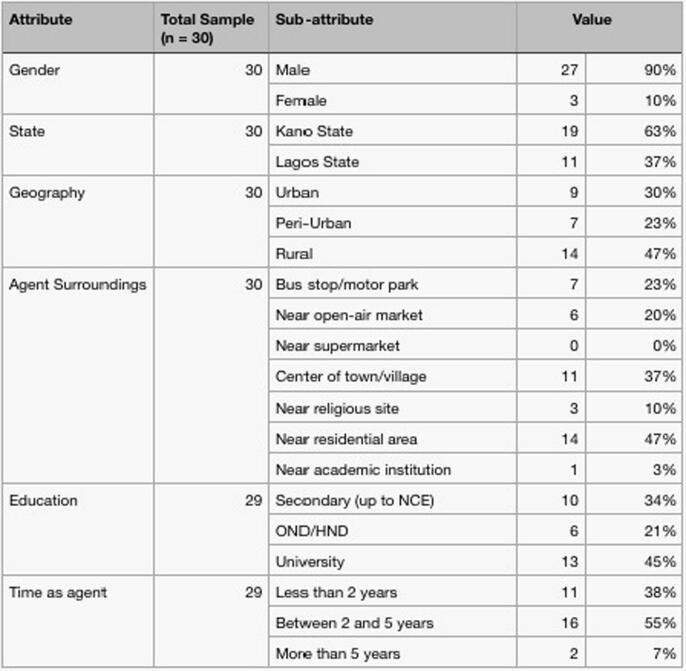
Demographics of the respondents.

The study adopted a qualitative approach using multiple cases to investigate the business models deployed by FSAs to explore opportunities for innovation and sustainability. The multiple case approach makes possible a comparative analysis of different business models of different agents and provides insight into how these models help deliver value propositions to customer segments (David-West, Iheanachor, et al., 2019). Berkowitz (1997) states that case studies provide very rich explorations of a subject or phenomenon as it develops in a real-world setting. Unlike the individual or intracase analysis approach that restricts the analysis to a single case, this study uses a cross-case analysis to analyse and synthesize data across the different cases. This allows for a comparative analysis of the different cases (Berkowitz, 1997, Cruzes et al., 2015, Mahoney, 1997; and Miles, Huberman, & Saldana, 2013) and provides evidence through multiple lenses (Eisenhardt, 1989), thus increasing the reliability of the findings. We purposively selected thirty active FSAs for the study. Using purposive sampling allowed us to focus on particular attributes of interest with a focus on agents’ BMI for sustainability at the bottom of the pyramid (BoP). The case selection criteria include operating location and model (dedicated versus nondedicated and interoperability across multiple service providers). We conducted semi-structured interviews with the principal operators of agent storefronts using a structured interview guide. We used interviews as they allowed us to understand the why behind the relationships As noted by Eisenhardt (1989) and business model practices of FSAs, Fig. 2 shows the demographic information of the sample. During the interviews, discussions using open-ended and theory-driven questions revolved around the drivers, elements and results of FSA business model innovation.

### 4.1 Data preparation and analysis

On completion of the agent interviews and operational observations, we created records of the agent responses and observation notes using a Microsoft Excel data entry template to capture theme codes. We created a coding tree comprising nodes from the two theoretical constructs and the business model canvas. The coding process was conducted in phases. In the first coding iteration, we used the autocode feature of NVivo that created nodes for each agent response. The coding process was iterative, moving back and forth between the data and the emerging code structure by identifying the most salient and frequently appearing codes across the interviews. This allowed comparison of codes among each response set to synthesize and explain larger segments of the data (Mathias, Huyghe, Frid, & Galloway, 2018) and detect patterns across the thematic areas.

Next, we reviewed the codable data of each agent case, tagging the components of the nodes. We used versus coding techniques to record comparisons among variables such as providers, service features, and channels. We used intercoder reliability techniques (Locke & Ramakrishna Velamuri, 2009) to validate the emerging codes and themes. After coding all 30 agent cases, we conducted node (axial) coding to identify the sub attributes of the business model components. Once we completed the coding, we carried out a pattern-wise aggregation of codes that we classified thematically using our a priori list of codes as a validation tool. For Osterwalder’s BMC, we confirmed nine broad themes with subthemes while for the SVCeB model, we confirmed four validated themes with underlying subthemes under each of the broad themes.

## 5 Results

### 5.1 Current agent business model of financial services

Our analysis shows that the FSAs do not have formalized business models and are reflected in the manner with which they conduct their businesses. However, they understand what is required to create and capture value both for their customers and the business. The data show that only 33% of the FSAs ran a dedicated model of provisioning financial services only while 67% were nondedicated. The data also show that approximately half of nondedicated agents operated in rural areas while the other half was split between urban and periurban locations.

### 5.2 Extent of business model innovation by FSAs

Our analysis shows that financial service agents are increasingly becoming innovative in their business models to be profitable and sustainable in delivering financial services to customers at the BoP. The data show that FSAs create value from different sources, including offering complementary services, devising novel means of serving their customers, building relationships that create customer lock-in and driving process efficiencies. We summarize the results using the business model canvas and sources of value creation in the e-business model (see Fig. 4, Fig. 5).

**Fig. 3:**
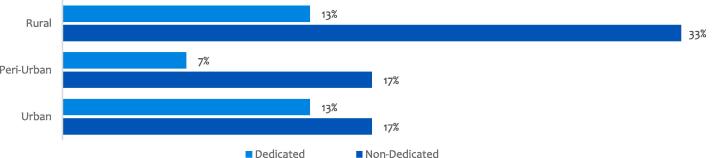
Agent business model typology.

**Fig. 4:**
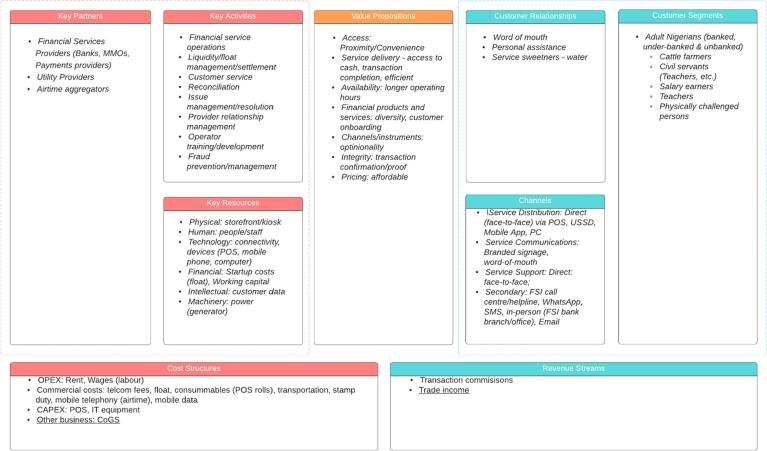
Business model canvas of FSAs.

**Fig. 5:**
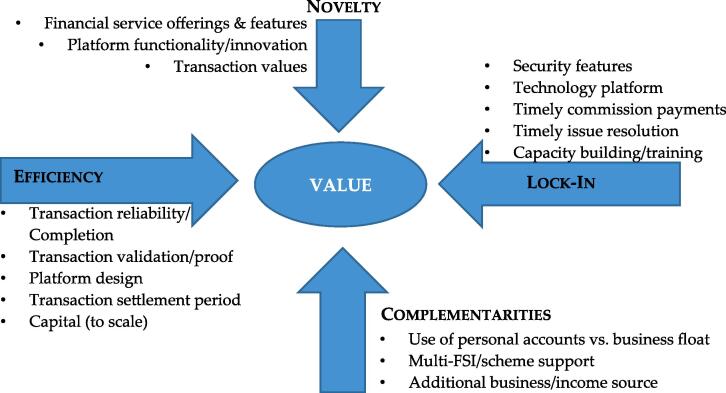
FSAs sources of value creation.

The business model canvas (see Fig. 3) highlights the key components deployed by agents to create and deliver value propositions to their customer segments.

### 5.3 Sources of value creation

We use Amit and Zott’s SVCeB model, as shown in Fig. 4, to illustrate the various sources of value creation among the agents operating the various business models.

## 6 Discussion

The results reveal some important themes and improve our overall understanding of the business models deployed by FSAs. Business model innovation typically occurs across various business model elements. We mapped these areas of BMI (see Fig. 3, Fig. 4) using Osterwalder and Pigneur’s BMC and Amit and Zott’s SVCeB. We discuss these areas of BMI below.

### 6.1 FSAs business model using the Osterwalder and Pigneur’s BMC

#### 6.1.1 Customer segments

FSAs exist to meet customer financial service needs. This pool of customers is diverse and cuts across cattle and small-holder farmers, civil servants, the larger salary earning population, and the physically challenged. To be profitable, FSAs must make deliberate efforts to increase their customer segments by becoming innovative in how they recruit and keep customers. However, our results show that the profiles of customers served by FSAs are typically the same across banked, underbanked, and unbanked customers. However, what is innovative about their customer segments is the location-specific advantage they exploit in citing their business where bank branches are not situated. This makes their value propositions of accessibility, convenience, availability and community trust very attractive to their customers. Other than the use of traditional below-the-line marketing strategies using word-of-mouth and service bundling to attract customers, customer retention is still a major challenge since the boutique of service offerings and quality are very much dependent on the FSPs they represent.

#### 6.1.2 Value proposition

FSA value propositions are limited in scope to those offered by the FSPs they represent. The term value proposition is a one-size-fits-all offering (David-West et al., 2018, Yunus et al., 2010). FSA value propositions can be grouped by access (bringing financial services directly to the customers) and service delivery (in terms of efficiency in completion). Although the value propositions deployed by the FSAs appear to be homogenous and predetermined by the FSPs they represent, these agents are deliberate in ensuring customer service despite challenges such as security and network connectivity. FSAs ensure that when their floats are low, they recycle funds from other businesses they operate to meet their financial service customers’ needs. We observed that there is significant room for an increase in FSA profitability if they extend the value propositions over and beyond the current scope of homogenous offerings.

#### 6.1.3 Channels

FSAs are traditionally last-mile operators and maintain and deploy customer touch points that ensure FSP-customer interaction. Dominant channels are in areas of service distribution and support (direct face-to-face, cards, POS, USSD, mobile apps and web); marketing (branded signage and word-of-mouth) and secondary customer support service channels (WhatsApp, SMS, email, in-person, phone calls). The quality of telecom infrastructure in the different locations was also observed to be a major factor in determining transaction success across various channels. In terms of marketing, FSAs relied solely on the customer outreach efforts of the FSPs they served. As Yunus et al. (2010) note, the way in which services are delivered to customers is critical to a business, and successful value delivery leverages outside networks and partners. While FSAs with the support of FSPs are doing well in this regard, there is significant room for innovation in exploring different ways of attracting new customers as FSAs have more frequent interactions with customers. FSAs can be innovative in using word-of-mouth and social media marketing as innovative means of consumer engagement and advertisement.

#### 6.1.4 Customer relationships

Johnson et al. (2008) note that it is often a relationship that makes the difference in a business and not necessarily resources. In light of this, FSAs essentially maintain face-to-face relationships with customers and, as a result, are able to build greater trust in their services. Although there are instances where agents communicated with their customers through phone calls and short message services, this was not widespread given the low literacy and low economic capabilities of their customer segments, which did not allow them to leverage digital interfaces for customer relationships. The use of airtime was also seen as cutting deep into their revenues; thus, they would rather wait until customers revisit their shops before redressing complaints that are within their means. While this is considered a prudent use of meagre resources, it limits their service efficiency. There is room for innovation in how customer relationships are maintained and extended as this holds potential for customer lock-in, trust building, repeat transactions, referrals, and profitability.

#### 6.1.5 Revenue streams

FSA revenues, as shown by this study, are mainly earned commissions and trade income from other business engagements. FSAs with other businesses were observed to have the potential to leverage their existing businesses to offer financial services at a lower cost. These were more positioned to access capital from their other businesses, leverage an existing customer base and technology and incurred no incremental costs for rent, utilities and maintenance. The existence of other businesses was also observed to help attract more customers, thus increasing their overall likelihood of profitability and sustainability. There is significant room for innovation in revenue diversification to provide liquidity and to reduce the associated costs and risks of long-distance travel to conventional commercial bank branches.

#### 6.1.6 Key activities

FSAs predominantly carry out activities such as cash-in, cash-out, recharge card vending, person-to-person transfers, bill payments, and account opening. These rather narrow scopes of activity are significantly determined by the FSPs. FSAs can aim at building innovative activities that support several other activities, such as customer awareness and financial literacy that would in turn increase customer numbers and transaction traffic. Other activities such as international transfers, loans, thrift/savings services, and micro insurance can be undertaken by FSAs as they hold the potential to create more robust value propositions for specific and important customer segments that are currently unserved.

#### 6.1.7 Cost structures

The cost structure of FSAs was observed to consist of start-up costs such as minimum liquidity; setting up the shop; technology acquisition (mobile phones, computer, POS, SIM); generator/solar panel; branding and marketing; and licensing fees. Recurring costs were observed to come from liquidity management, staff/employees (labour), power generators, rent, Internet/data, water, refuse, and maintenance. An agent’s profitability was a function of these cost drivers and varied by the particular constraints faced by an agent and the approaches to cost management.

Nondedicated agents were observed to have lower marginal costs and typically break even more quickly as transaction volumes increase. A higher proportion of costs were observed to be fixed and resulted in high dependence of the agent’s profitability on transaction volume. Liquidity management costs are also higher and vary with distance from commercial banks. There are several innovation opportunities that can in turn enhance agent profitability. These include making fewer trips to the bank, increasing transaction volumes, and increasing the size of the support businesses that reduce liquidity costs and provide additional working capital for the agency business.

#### 6.1.8 Key resources

The key resources deployed by FSAs in their delivery of digital financial services include mobile phones, laptops, cash, office space, working capital, POS, website, and personnel. A very important resource is working capital, which is enhanced in the presence of a counterpart business that helps generate additional float and reduces liquidity pressure that may come from heavy withdrawals by customers. In this regard, there is significant room for innovation. FSAs can deploy an assortment of approaches in enhancing agent liquidity and increasing overall profitability. These will include maintaining a database of customers and brand visibility.

#### 6.1.9 Key partners

The FSAs in the study had significant partnerships with entities such as technology providers, commercial banks, and mobile network operators. The partners are critical in the provision of key resources and in driving core FSA activities. Given the small scale of agents’ operations and their significantly lower bargaining power, aggregating their demand through their national association can help create a major bargaining group that can help members negotiate better prices from the services offered to them by these key partners and help drive innovation at a larger scale. These negotiations can result in significantly lower costs and foster innovation in how they define and manage their relationships with their key partners.

### 6.2 FSAs’ sources of value creation using Amit and Zott’s SVCeB

#### 6.2.1 Efficiency

There was a significant level of efficiency among some of the studied FSAs. By leveraging existing businesses (business centres, provision stores, airtime vending etc.) to deliver financial services, the FSAs achieved some level of efficiency in terms of service availability and resource utilization. However, this was limited to nondedicated agents (i.e., FSAs conducting business other than financial services). Dedicated agents were less efficient in the use of resources, and this was challenging to increase their profitability as they also had to off-set their capital and operational costs. Although the FSA guideline requires them to be owners of existing businesses, the study revealed that a significant number of agents operated only financial service businesses.

#### 6.2.2 Complementarity

Agents who were nondedicated were able to cross-sell other products that are not financial services to customers patronizing their other businesses. For instance, customers buying household utilities and bill payments such as electricity and cable TV also utilized commission-based financial services at the agent locations, thereby increasing the agents’ efficiency to scale, grow revenue and become profitable. This phenomenon also helps customers reduce travel time to conduct several other transactions in different locations since they can now access them at the agent location. However, dedicated agents have a customer segment that is limited to financial service users who must visit other vendors to conduct several other transactions. FSAs that are nonexclusive (i.e., those serving multiple FSPs) also leverage the same technology devices, rent, power, and other cost elements to serve customers with different financial products, thus expanding their customer reach at low operational and fixed costs. Although complementarity would require service bundling, FSAs’ ability to innovate their business models at this level was limited as the suites of financial services they offered were largely determined by the FSPs.

#### 6.2.3 Novelty

The study showed that all FSAs depended on the FSPs they represent to drive innovation. For instance, in cases where branding was not provided by the FSPs, the FSAs had no signage indicating the presence of the financial service business at their location but depended solely on word-of-mouth or referrals, which were largely absent. Although novelty drives complementarity and efficiency, the low levels of transactions resulting from low DFS adoption at the BoP meant that the agents struggled with limited resources to be innovative. Furthermore, the assets, resources and capabilities (finance, digital capability, marketing skills) required to create novel service delivery mechanisms were largely lacking.

## 7 Conclusion

Of the three business model innovation types identified by Giesen et al. (2007), FSAs typically fit within *revenue business model innovation,* which refers to how firms generate value. However, the nature of FSA businesses places them as financial services intermediaries with products and services that cannot be altered at the agent level. FSAs deliver services that help resolve social market failures such as lack of access to financial services, and in doing so, they stimulate opportunities to add social value to lives and communities. However, FSAs require business models that help them achieve business sustainability and social impact while managing social and business tensions. The issue of how tensions between commercial and social market failures can be resolved is still a key gap in the literature (Margiono, Zolin, & Chang, 2018). Thus, the sustainability of FSAs requires that they innovate their activities along pathways that can grow their revenues across each of the business model component dimensions.

Although suboptimal, we identified three business models deployed by FSAs to create and deliver value. The first is an exclusive versus nonexclusive model, the second is a dedicated versus nondedicated model, and the third is a single-location versus multiple-location model. Agents who opt for any nondedicated, nonexclusive, and multilocation models or a combination of these models are more innovative and better positioned to be profitable and sustainable than those who adopt any model(s) among the single location, exclusive or dedicated models. While the former models may drive agents’ businesses in the moment, the weakness in these models is that there is a limit to the extent to which FSAs can innovate as the services they drive are designed by their FSPs. The different levers that can be pulled by FSAs along the dimensions of the business model as defined by Osterwalder and Pigneur (2010) show inefficiencies in the current FSA business models. As Yunus et al. (2010) indicate, social businesses such as FSAs may not be able to replicate the conventional for-profit business model. However, they must carry out BMI (Johnson et al., 2008) within ethical limits to guarantee their long-term sustainability.

This study reveals that although there are some observed elements of BMI displayed by the FSAs in our sample, they may be unable to profitably and sustainably serve their customers over the long term. Although the agents have developed their business models along the lines of direct dedication to financial services operations or nondedication, the study reveals that there is still significant room for innovation in various aspects of their business models. Most of the deployed business models are a consequence of their position in the ecosystem, their bargaining powers as very small businesses and the existing terms and conditions dictated by the host financial service providers represented by the FSAs. This study is a first important step in contributing to the knowledge of why FSAs in Nigeria seldom innovate their business models.
